# Geometric Construction of Eighth-Order Optimal Families of Ostrowski's Method

**DOI:** 10.1155/2015/614612

**Published:** 2015-03-26

**Authors:** Ramandeep Behl, S. S. Motsa

**Affiliations:** School of Mathematics, Statistics and Computer Science, University of KwaZulu-Natal, Private Bag X01, Scottsville, Pietermaritzburg 3209, South Africa

## Abstract

Based on well-known fourth-order Ostrowski's method, we proposed many new interesting optimal families of eighth-order multipoint methods without memory for obtaining simple roots. Its geometric construction consists in approximating *f*_*n*_′ at *z_n_* in such a way that its average with the known tangent slopes *f*_*n*_′ at *x_n_* and *y_n_* is the same as the known weighted average of secant slopes and then we apply weight function approach. The adaptation of this strategy increases the convergence order of Ostrowski's method from four to eight and its efficiency index from 1.587 to 1.682. Finally, a number of numerical examples are also proposed to illustrate their accuracy by comparing them with the new existing optimal eighth-order methods available in the literature. It is found that they are very useful in high precision computations. Further, it is also noted that larger basins of attraction belong to our methods although the other methods are slow and have darker basins while some of the methods are too sensitive upon the choice of the initial value.

## 1. Introduction

Multipoint iterative methods for solving nonlinear equation,

(1) $$\begin{array}{c} f\left(x\right)=0, \end{array}$$

have drawn a considerable attention in the first decade of the 21st century, which led to the construction of many methods of this type. These methods are primarily introduced with the aim to achieve as high as possible order of convergence using a fixed number of function evaluations. However, multipoint methods do not use higher order derivatives and have great practical importance since they overcome the theoretical limitations of one-point methods regarding their convergence order and computational efficiency.

As the order of an iterative method increases, so does the number of functional evaluations per step. The efficiency index [1] gives a measure of the balance between those quantities, according to the formula *p* ^1/*d*^, where *p* is the order of convergence of the method and *d* is the number of functional evaluations per step. According to the Kung-Traub conjecture [2], the order of convergence of any multipoint method cannot exceed the bound 2^*n*−1^, called the optimal order. Thus, the optimal order for a method with three functional evaluations per step would be four. The well-known King's family of methods [3] is an example of fourth order multipoint methods requiring three functional evaluations per full iteration, which is given by

(2) yn=xn−f(xn)f′(xn),xn+1=xn−fxn2+(β−1)f(xn)f(yn)+βfyn2f′xnfxn+β−2fyn,iiiiiiiiiiiiiiiiiiiiiiiiiiiiiiiiiiiiiiiiiiiiiiiiiiiiiiiiiiwhere  β∈R.

For *β* = 0, one can easily get the well-known Ostrowski's method. From practical point of view, King's family [3] and Ostrowski's method [1, 4] are one of the most efficient multipoint fourth-order methods known to date because they have simple body structures and do not require the computation of a second-order derivative. They have efficiency index equal to 1.5874, which is very competitive.

In recent years, based on the King's method and Ostrowski's method, some higher order iterative methods have been proposed and analyzed for solving nonlinear equations. J. R. Sharma and R. Sharma [5] proposed a family of Ostrowski's method with eighth-order convergence, which is given by

(3) yn=xn−f(xn)f′(xn),zn=yn−f(xn)f(yn)f′(xn)(f(xn)−2f(yn)),xn+1=zn−f(zn)f′(xn)H(μn),

where *μ* = *f*(*y*_*n*_)/*f*(*x*_*n*_) and *H*(*t*) represents a real-valued function with *H*(0) = 1, *H*′(0) = 2, and |*H*′′(0)|<*∞*. We will refer to this method as SSM_8_.

Liu and Wang [6] have also presented another eighth-order family of Ostrowski's method, requiring three-function and one-derivative evaluation per iteration:

(4) yn=xn−f(xn)f′(xn),zn=yn−f(xn)f(yn)f′(xn)(f(xn)−2f(yn)),xn+1=zn−fznf′xnfxn−fynfxn−2fyn24f(zn)f(xn)+bf(zn)hhhhhhhhhhh+f(zn)(f(yn)−af(zn))hhhhhhhhhhh(f(xn)−f(yn))(f(xn)−2f(yn))2+4f(zn)f(xn)+bf(zn),

where *a* and *b* are two free disposable parameters. We will refer to this method as LWM_8_.

Soleymani et al. [7] also proposed eighth-order variant of Ostrowski's method, which is given by

(5) yn=xn−f(xn)f′(xn),zn=yn−f(xn)f(yn)f′(xn)(f(xn)−2f(yn)),xn+1=zn−f(zn)f′(xn)f(xn)(f(xn)−2f(yn))×1+2f(zn)f(xn)+f(zn)f(yn)+f(yn)f(xn)2hhh+ 2f(yn)f(xn)3−f(zn)f′(xn)+f(yn)f′(xn)3.

We will refer to this method as SM_8_.

The main goal of this paper is to develop a general class of very efficient three-point methods for solving nonlinear equations. Here, we derived several new optimal families of eighth-order Ostrowski's method by taking the arithmetic mean of three slopes and then applying weight function approach. In terms of computational cost, they require four functional evaluations per iteration. Thus, the new family adds only one evaluation of the function at another point other than Ostrowski's method and order increases from four to eight. This property of the new methods provides a new example of multipoint methods without memory having optimal order of convergence. The efficiency of the methods is tested on a number of numerical examples.

## 2. Development of Optimal Eighth-Order Families of Ostrowski's Method

Newton's method is probably the best known and most widely used one-point iterative method for solving nonlinear equation (1). It converges quadratically to a simple root and linearly to a multiple root. Its geometric construction consists in considering the straight line

(6) $$\begin{array}{c} y=ax+b, \end{array}$$

then determining the unknowns *a* and *b* by imposing the tangency conditions:

(7) $$\begin{array}{c} y\left(x_{n}\right)=f\left(x_{n}\right),\;\;y^{\prime }\left(x_{n}\right)=f^{\prime }\left(x_{n}\right), \end{array}$$

and thereby obtaining the tangent line

(8) $$\begin{array}{c} y\left(x\right)-f\left(x_{n}\right)=f^{\prime }\left(x_{n}\right)\left(x-x_{n}\right), \end{array}$$

to the graph of *f*(*x*) at (*x*_*n*_, *f*(*x*_*n*_)).

The point of intersection of this tangent line with *x*-axis gives the celebrated Newton's method

(9) $$\begin{array}{c} x_{n+1}=x_{n}-\frac{f\left(x_{n}\right)}{f^{\prime }\left(x_{n}\right)},\;n\geq 0. \end{array}$$

The convergence order and computational efficiency of the one-point iterative methods are lower than multipoint iterative methods [8] because multipoint iterative methods can overcome theoretical limits of one-point methods concerning the convergence order and computational efficiency. In recent years, many multipoint iterative methods have been proposed that improve the local convergence order of the classical Newton's method. In 1973, King [3] had considered the following fourth-order iteration scheme:

(10) yn=xn−f(xn)f′(xn),xn+1=yn−f(yn)f′(yn), n=0,1,2,….

But according to the Kung-Traub conjecture [2], the above scheme (10) is not an optimal method because it has fourth-order convergence and requires four functional evaluations per full iteration. However, King [3] had reduced the number of function evaluations by using some suitable approximation of *f*′(*y*_*n*_). In fact, King had taken the approximation of *f*′(*y*_*n*_) in such a way that its average with the known tangent slopes *f*_*n*_′ at *x*_*n*_ and *y*_*n*_ is the same as the known secant slopes; that is,

(11) $$\begin{array}{c} \frac{f^{\prime }\left(y_{n}\right)+f^{\prime }\left(x_{n}\right)}{2}=\frac{f\left(x_{n}\right)-f\left(y_{n}\right)}{x_{n}-y_{n}}. \end{array}$$

After solving (11), one can get the following value of *f*′(*y*_*n*_) as

(12) $$\begin{array}{c} f^{\prime }\left(y_{n}\right)=\frac{f^{\prime }\left(x_{n}\right)\left(f\left(x_{n}\right)-2f\left(y_{n}\right)\right)}{f\left(x_{n}\right)}. \end{array}$$

Using this value in scheme (10), we get

(13) yn=xn−f(xn)f′(xn),xn+1=yn−f(xn)f(yn)f′(xn)(f(xn)−2f(yn)), n=0,1,2,3,….

This is well-known Ostrowski's method [1, 4]. It is very interesting to note that, by adding one evaluation of the function at another point iterated by Newton's method, the order of convergence increases from two to four and is free from the second-order derivative.

Now, we intend to derive the new optimal eighth-order family of Ostrowski's method. For this, we consider a three-step iteration scheme with existing Ostrowski's method as follows:

(14) yn=xn−f(xn)f′(xn),zn=yn−f(xn)f(yn)f(xn)−2f(yn),xn+1=zn−f(zn)f′(zn).

Again the above method is not optimal according to the Kung-Traub conjecture [2], because it has eighth-order convergence and requires five functional evaluations per full iteration. However, we can reduce the number of function evaluations by using some suitable approximation of *f*′(*z*_*n*_). In fact, we will take the approximation of *f*′(*z*_*n*_) similar to King's approximation in such a way that its average with the known slopes *f*_*n*_′ at *x*_*n*_, *y*_*n*_, and *z*_*n*_ is the same as the known weighted average of secant slopes:

(15) f′(yn)+f′(xn)+f′(zn)3  =132f(xn)−f(yn)xn−yn+f(zn)−f(yn)zn−yn.

After solving (15), we get

(16) $$\begin{array}{c} f^{\prime }\left(z_{n}\right)=\frac{f^{\prime }\left(x_{n}\right)\left(f\left(x_{n}\right)-2f\left(y_{n}\right)\right)\left(f\left(y_{n}\right)-f\left(z_{n}\right)\right)}{f\left(x_{n}\right)f\left(y_{n}\right)}. \end{array}$$

Using this value of *f*′(*z*_*n*_) in scheme (14), we get

(17) yn=xn−f(xn)f′(xn),zn=yn−f(xn)f(yn)f′(xn)(f(xn)−2f(yn)),xn+1=zn−f(xn)f(yn)f(zn)f′(xn)(f(xn)−2f(yn))(f(yn)−f(zn)).

This is a new sixth-order Ostrowski's method. It satisfies the following error equation:

(18) $$\begin{array}{c} e_{n+1}=\left(c_{2}^{5}-c_{2}^{3}c_{3}\right)e_{n}^{6}+O\left(e_{n}^{7}\right), \end{array}$$

where *e*_*n*_ = *x*_*n*_ − *r* and *c*_*k*_ = (1/*k*!)(*f* ^(*k*)^(*r*)/*f*′(*r*)), *k* = 2,3,….

Again, the above method is not optimal according to the Kung-Traub conjecture [2]. Therefore, to improve the order of convergence of this method, we will now make use of weight function approach to build our optimal families of this iterative method by a simple change in its third step. Therefore, we consider

(19) yn=xn−f(xn)f′(xn),zn=yn−f(xn)f(yn)f′(xn)(f(xn)−2f(yn)),xn+1 =zn−fxnfynfznf′xnfxn−2fynfyn−fzn  ×Q(u,v),

where *u* = *f*(*z*_*n*_)/*f*(*x*_*n*_), *v* = *f*(*y*_*n*_)/*f*(*x*_*n*_), and *Q*(*u*, *v*) is a two variable real-valued weight function such that its order of convergence reaches at the optimal level eight without using any more functional evaluations.  Theorem 1 indicates that under what conditions on the weight function in (19) the order of convergence will reach at the optimal level eight.

## 3. Order of Convergence

**Theorem 1 .**

Let a sufficiently smooth function *f* : *D*⊆*ℝ* → *ℝ* have a simple zero *r* in the open interval *D*. Let *Q*(*u*, *v*) be a two-variable real-valued differentiable function. If an initial approximation *x*_0_ is sufficiently close to the required root *r* of a function *f*, then the convergence order of the family of three-point methods (19) is equal to eight when it satisfies the following conditions:

(20) $$\begin{array}{c} Q_{00}=1,\;\;Q_{10}=2,\;\;Q_{01}=0,\\ Q_{02}=2,\;\;Q_{03}=12, \end{array}$$

where *Q*_*ij*_ = (1/*i*!*j*!)(∂*Q*(*u*, *v*)/∂*u* ^*i*^ *v* ^*j*^)|_(0,0)_, for *i* = 0,1, 2,3 and *j* = 0,1, 2,3.

It satisfies the following error equation:

(21) en+1=−c22c22−c3×−7+Q11c23−−4+Q11c2c3−c4e8+Oe9,

where *e*_*n*_ and *c*_*k*_ are already defined in (18).

**Proof**

Let *x* = *r* be a simple zero of *f*(*x*). Expanding *f*(*x*_*n*_) and *f*′(*x*_*n*_) about *x* = *r* by the Taylor's series expansion, we have

(22) fxn=f′(r)×en+c2en2+c3en3+c4en4+c5en5hhh+ c6en6+c7en7+c8en8+O(en9),f′xn=f′(r)×1+2c2en+3c3en2+4c4en3+5c5en4hhh+ 6c6en5+7c7en6+8c8en7+O(en9),

respectively.

From (22), we have

(23) f(xn)f′(xn)=en−c2en2+2c22−c3en3+−4c23+7c2c3−3c4en4+O(en5),

and in combination with the Taylor series expansion of *f*(*x*_*n*_ − (*f*(*x*_*n*_)/*f*′(*x*_*n*_))) about *x* = *r*, we have

(24) f(yn)=fxn−fxnf′xn=f′(r)×c2en2+−2c22+2c3en3hhh+5c23−7c2c3+3c4en4hhh−26c24−12c22c3+3c32+5c2c4−2c5en5+O(en6).

Therefore, we have

(25) fxnfxn−2fyn =1+2c2en+−2c22+4c3en2  +−4c2c3+6c4en3+4c24−6c22c3−4c2c4+8c5en4  −24c25−14c23c3+5c22c4  hhhh−2c3c4+c29c32+2c5−5c6en5+O(en6),un=f(yn)f′(xn)=c2en2+−4c22+2c3en3+13c23−14c2c3+3c4en4−2(19c24−32c22c3+6c32+10c2c4−2c5)en5+O(en6).

From (25), we have

(26) zn=yn−f(xn)f′(xn)f(yn)f(xn)−2f(yn),=c23−c2c3en4−22c24−4c22c3+c32+c2c4en5+10c25−30c23c3+12c22c4−7c3c4+3c26c32−c5×en6+O(en7).

Now, expanding *f*(*z*_*n*_) about *r*, we get

(27) fzn=f′(r)×c23−c2c3en4−22c24−4c22c3+c32+c2c4en5+3c26c32−c5en6hhh+10c25−30c23c3+12c22c4−7c3c4+3c26c32−c5en6hhhhhhhhh+ 3c26c32−c5en6+O(en7).

Furthermore, we have

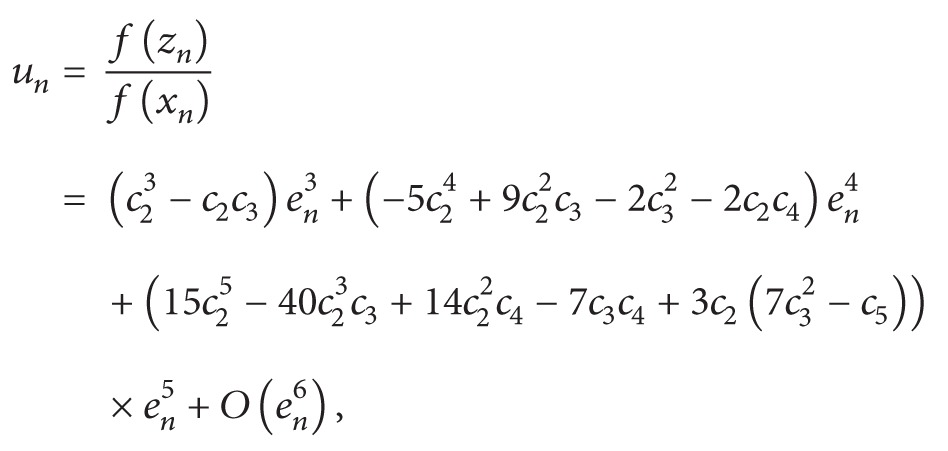
(28)

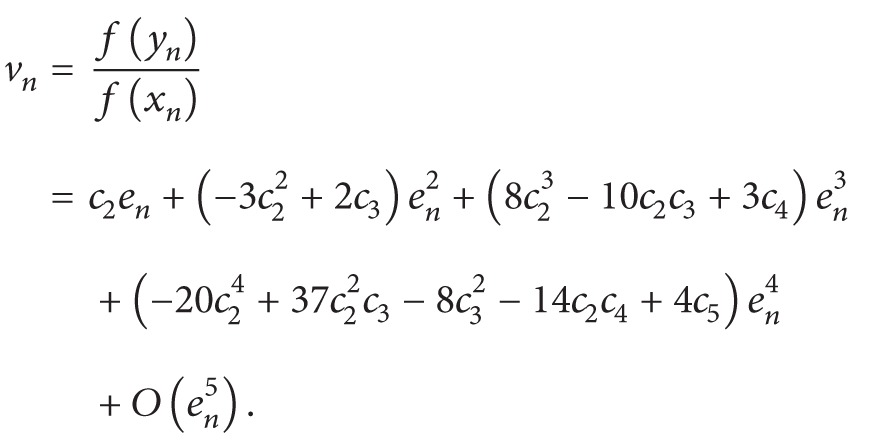
(29)
Since it is clear from (29) that *u*_*n*_ and *v*_*n*_ are of order *e*_*n*_^3^ and *e*_*n*_ respectively, therefore, we can expand weight function *Q*(*u*, *v*) in the neighborhood of origin by Taylor series expansion up to third-order terms as follows:

(30) Qu,v=Q00+Q10u+Q01v+12Q20u2+2Q11uv+Q02v2+16(Q30u3+3Q21u2v+3Q12uv2+Q03v3).

Using (28), (29), and (30) in scheme (19), we have the following error equation size:

(31) en+1 =zn−fxnfynfznf′xnfxn−2fynfyn−fzn  ×Q(u,v)=−(−1+Q00)c2c22−c3en4  +(−4+4Q00−Q01)c24  hhh+8−8Q00+Q01c22c3  hhh+2(−1+Q00)c32+2(−1+Q00)c2c4en5  +10−9Q00−Q022+7Q01c25  hhh+12(−60+58Q00+Q02−26Q01)c23c3  hhh+2(6−6Q00+Q01)c22c4+7(−1+Q00)c3c4  hhh+c218−18Q00+4Q01c32hhhhhhhhhhh10−9Q00−Q022+7Q01c25+3(−1+Q00)c518−18Q00+4Q01c32en6  +−20+14Q00+5Q02−Q036−Q10−29Q01c26  hhh−16−480+408Q00+54Q02−Q03−12Q10hhhhhhhhhhh−474Q01c24c3  hhh+(−40+38Q00+Q02−21Q01)c23c4  hhh+c22(−80+76Q00+3Q02−Q10−50Q01)c32hhhhhhhhhhh(−80+76Q00+3Q02−Q10−50Q01)c32+(16−16Q00+3Q01)c5  hhh+2(6−6Q00+2Q01)c33+3(−1+Q00)c42hhhhhhhhhhh+ 5(−1+Q00)c3c5(6−6Q00+2Q01)c33+3(−1+Q00)c42hhhhhhhhhhh+c22(26−26Q00+7Q01)c3c4hhhhhhhhhhhhhh+ 4(−1+Q00)c6Q036en7  +16216−90Q00−174Q02+13Q03+54Q10hhhhhhhh−6Q11+558Q01c27hhhhhhhh+−1068+690Q00+450Q02−23Q03hhhhhhhhhhh−156Q10+12Q11−2004Q01c25c3hhhhhhhh+2303−246Q00−45Q02+Q03hhhhhhhhhhhh+12Q10+348Q01c24c4hhhhhhhh+c23−2−756+624Q00+147Q02−4Q03hhhhhhhhhhhhhhhhh−63Q10+3Q11−987Q01c32hhhhhhhhhhhhh+3(−102+96Q00+3Q02−58Q01)c5hhhhhhhh+3c22−418+390Q00+21Q02hhhhhhhhhhhhhhh− 8Q10−310Q01c3c4hhhhhhhhhhhhhh+ 8(5−5Q00+Q01)c6hhhhhhhh−610(−5+5Q00−2Q01)c32c4hhhhhhhhhhhh−17(−1+Q00)c4c5hhhhhhhhhhhh−13(−1+Q00)c3c6hhhhhhhh+6c2−91+87Q00+6Q02hhhhhhhhhhhhhhh−4Q10−76Q01c33hhhhhhhhhhhhh+(37−37Q00+12Q01)c42hhhhhhhhhhhhh+4(17−17Q00+5Q01)c3c5hhhhhhhhhhhhh+ 5(−1+Q00)c7en8+O(en9).

For obtaining an iterative method of order eight, the coefficients of *e*_*n*_^4^, *e*_*n*_^5^, *e*_*n*_^6^, and *e*_*n*_^7^ in the error equation (31) must be zero simultaneously. After simplifications, we have the following equations involving *Q*_00_, *Q*_10_, *Q*_01_, *Q*_02_, and *Q*_03_,

(32) $$\begin{array}{c} \left(-1+Q_{00}\right)=0,\\ \left(-4+4Q_{00}-Q_{01}\right)=0,\\ \left(8-8Q_{00}+Q_{01}\right)=0,\\ \left(10-9Q_{00}-\frac{Q_{02}}{2}+7Q_{01}\right)=0,\\ \left(-60+58Q_{00}+Q_{02}-26Q_{01}\right)=0,\\ \left(6-6Q_{00}+Q_{01}\right)=0,\\ \left(18-18Q_{00}+4Q_{01}\right)=0,\\ \left(-20+14Q_{00}+5Q_{02}-\frac{Q_{03}}{6}-Q_{10}-29Q_{01}\right)=0,\\ \left(-480+408Q_{00}+54Q_{02}-Q_{03}-12Q_{10}-474Q_{01}\right)=0,\\ \left(-40+38Q_{00}+Q_{02}-21Q_{01}\right)=0,\\ \left(-80+76Q_{00}+3Q_{02}-Q_{10}-50Q_{01}\right)=0,\\ \left(16-16Q_{00}+3Q_{01}\right)=0,\\ \left(6-6Q_{00}+2Q_{01}\right)=0,\\ \left(26-26Q_{00}+7Q_{01}\right)=0. \end{array}$$

After simplifying (32), we have the following conditions on the weight function:

(33) $$\begin{array}{c} Q_{00}=1,\;\;Q_{10}=2,\;\;Q_{01}=0,\\ Q_{02}=2,\;\;Q_{03}=12. \end{array}$$

Finally, we get the following error equation:

(34) en+1=−c22c22−c3×−7+Q11c23−−4+Q11c2c3−c4en8+O(en9).

This reveals that the three-step class of Ostrowski's method (19) reaches the optimal order of convergence eight by using only four functional evaluations per full iteration. This completes the proof of the Theorem 1.

## 4. Special Cases

In this section, we will consider some particular cases of the proposed scheme (19) depending upon the weight function *Q*(*u*, *v*) as follows.

**Case 1 .**

Let us consider the following weight function:

(35) $$\begin{array}{c} Q\left(u,v\right)=\left(au+1\right)v^{2}+2u+2v^{3}+1. \end{array}$$

It can be easily seen that the abovementioned weight function *Q*(*u*, *v*) satisfies all the conditions of Theorem 1. Therefore, we obtain a new optimal family of eighth-order methods given by

(36) yn=xn−f(xn)f′(xn),zn=yn−f(xn)f(yn)f′(xn)(f(xn)−2f(yn)),xn+1=zn−fynfzn+fxn2fxn+2fznhhhhh×2fyn3+fyn2+fxn2fxn+2fznhhhhhhhh×fxn+afznhhhhhhhh+ fxn2fxn+2fzn×f′xnfxn2fxn−2fynhhh×fyn−fznf′xnfxn21/2.

**Case 2 .**

Let us consider the following weight function:

(37) $$\begin{array}{c} Q\left(u,v\right)=1-\frac{v}{2}+\frac{4bu+bv}{2b-2u-4bv+4uv}. \end{array}$$

It can be easily seen that the abovementioned weight function *Q*(*u*, *v*) satisfies all the conditions of Theorem 1. Therefore, we obtain a new optimal family of eighth-order method given by

(38) yn=xn−f(xn)f′(xn),zn=yn−f(xn)f(yn)f′(xn)(f(xn)−2f(yn)),xn+1=zn−fxnfynfzn−bfxnfyn+4fzn22fyn−fxnbfxn−fznhhhhh×1−fyn2fxnhhhhhhhhh−bfxnfyn+4fznhhhhhhhhh×22fyn−fxnhhhhhhhhhhhhfxnfynfzn×bfxn−fzn1/21−fyn2fxn×f′xn−2fyn+fxnhhhf′xn−2fyn+fxn×fyn−fzn1/2.

**Case 3 .**

Let us consider the following weight function:

(39) $$\begin{array}{c} Q\left(u,v\right)=\frac{3}{4}-\frac{v}{2}+\frac{b+8bu}{4b-8bv-4uv+8uv^{2}}. \end{array}$$

It can be easily seen that the abovementioned weight function *Q*(*u*, *v*) satisfies all the conditions of Theorem 1. Therefore, we obtain a new optimal family of eighth-order method given by

(40) yn=xn−f(xn)f′(xn),zn=yn−f(xn)f(yn)f′(xn)(f(xn)−2f(yn)),xn+1 =zn−f(yn)f(zn)  ×4f′xn−2fyn+fxn2fyn−fzn  hhh×−bfxn2+fynfzn1/2  ×fyn4fyn2−8fynfxn+3fxn2hhhhhh×f(zn)−4bfxn2hhhhhh×fyn2−2fynfxnhhhhhhhhhfyn4fyn2−8fynfxn+3fxn2+f(xn)(f(xn)+2f(zn)).

It is straightforward to see that per step all the proposed family of methods require four functional evaluation, namely, *f*(*x*_*n*_), *f*(*y*_*n*_)*f*(*z*_*n*_), and *f*′(*x*_*n*_). In order to obtain an assessment of the efficiency of our proposed methods, one will make use of efficiency index [1]. For newly proposed eighth-order three-point methods, one finds *p* = 8 and *d* = 4 to get $E=\sqrt[{4}]{8}\approx 1.682$ which is better than $E=\sqrt{2}\approx 1.414$, the efficiency index of Newton's method. Further, by choosing different kinds of weight functions one can develope several new optimal families of eight-order multipoint methods.

## 5. Numerical Experiments

In this section, we will check the effectiveness of the new optimal methods. We employ the present methods (38) (for *b* = −1/2) and (40) (for *b* = −1/4) denoted by MOM1_8_ and MOM2_8_, respectively, to solve nonlinear equations given in Table 1. We compare them with J. R. Sharma and R. Sharma method (SSM_8_), Liu and Wang method (LWM_8_), Thukral method [9] (TM_8_), and Soleymani method (SM_8_), respectively. For better comparisons of our proposed methods, we have given two comparison tables in each example: one is corresponding to absolute error value of given nonlinear functions (with the same total number of functional evaluations = 12) and the other is with respect to number of iterations taken by each method to obtain the root correct up to 35 significant digits in Tables 2 and 3, respectively. All computations have been performed using the programming package *Mathematica* 9 with multiple precision arithmetic. We use *ϵ* = 10^−34^ as a tolerance error. The following stopping criteria are used for computer programs:

- |*x*_*n*+1_ − *x*_*n*_ | <*ϵ*,
- |*f*(*x*_*n*+1_)|<*ϵ*.

## 6. Attractor Basins in the Complex Plane

We here investigate the comparison of the attained multiple root finders in the complex plane using basins of attraction. It is known that the corresponding fractal of an iterative root-finding method is a boundary set in the complex plane, which is characterized by the iterative method applied to a fixed polynomial *p*(*z*) ∈ *ℂ*; see, for example, [10, 11]. The aim herein is to use basin of attraction as another way for comparing the iteration algorithms.

From the dynamical point of view, we consider a rectangle *D* = [−3,3]×[−3,3] ∈ *ℂ* with a 400 × 400 grid, and we assign a color to each point *z*_0_ ∈ *D* according to the multiple root at which the corresponding iterative method starting from *z*_0_ converges, and we mark the point as black if the method does not converge. In this section, we consider the stopping criterion for convergence to be less than 10^−4^ wherein the maximum number of full cycles for each method is considered to be 200. In this way, we distinguish the attraction basins by their colors for different methods.

**Figure 1:**
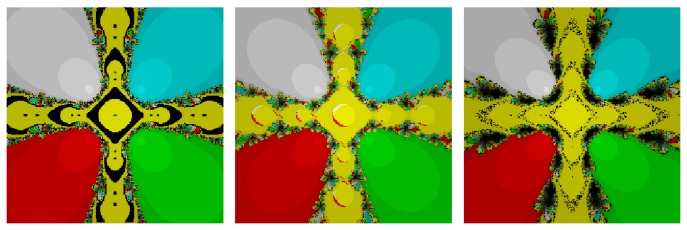
The basins of attraction for SSM_8_, LW_8_, and TM_8_, respectively, in problem 1.

**Figure 2:**
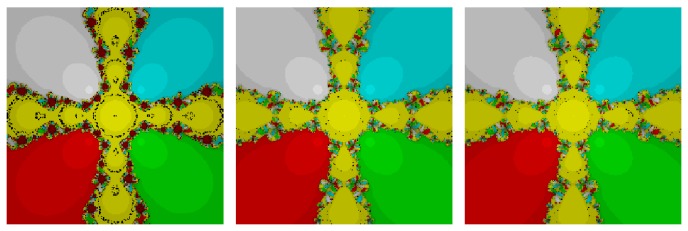
The basins of attraction for SM_8_, OM_8_^1^, and OM_8_^2^, respectively, in problem 1.

*Test Problem 1*. Let *p*_1_(*z*) = (*z* ^5^ + *z*), having simple zeros {−0.707107 − 0.707107*i*, −0.707107 + 0.707107*i*, 0, 0.707107 − 0.707107*i*, 0.707107 + 0.707107*i*}. It is straight forward to see from Figures 1 and 2 that our methods, namely, OM_8_^1^ and OM_8_^2^, contain lesser number of divergent points in comparison to the methods, namely, SSM_8_, LW_8_, and TM_8_. Further, our methods have also less chaotic behavior than other methods, namely, LW_8_^1^ and SM_8_^2^. 

**Figure 3:**
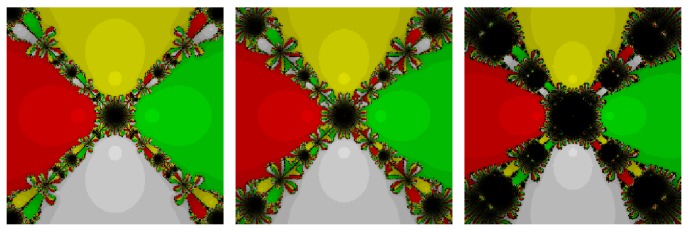
The basins of attraction for SSM_8_, LW_8_, and TM_8_, respectively, in problem 2.

**Figure 4:**
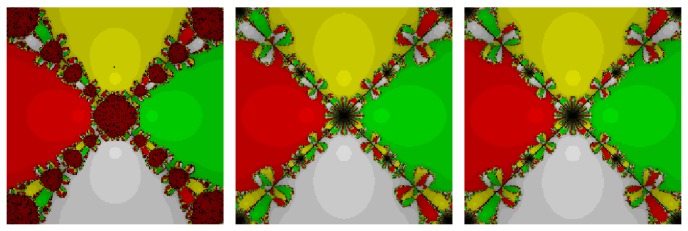
The basins of attraction for SM_8_, OM_8_^1^, and OM_8_^2^, respectively, in problem 2.

*Test Problem 2*. Let *p*_2_(*z*) = (*z* ^4^ − 1), having simple zeros {−1, −*i*, *i*, 1}. It is straight forward to see from Figures 3 and 4 that our method, namely, OM_8_^1^ and OM_8_^2^, performed better and larger basins of attraction as compared to the other methods, namely, SSM_8_, LW_8_, TM_8_, and SM_8_. Further, our methods have lesser number of divergent points and less chaotic behavior in comparison with other methods. 

**Figure 5:**
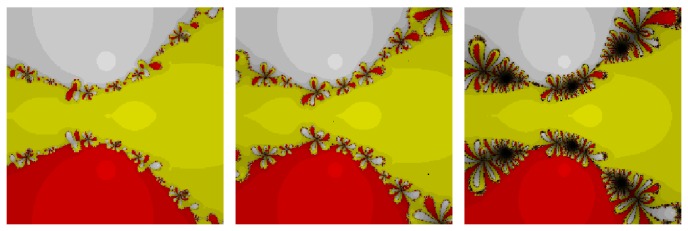
The basins of attraction for SSM_8_, LW_8_, and TM_8_, respectively, in problem 3.

**Figure 6:**
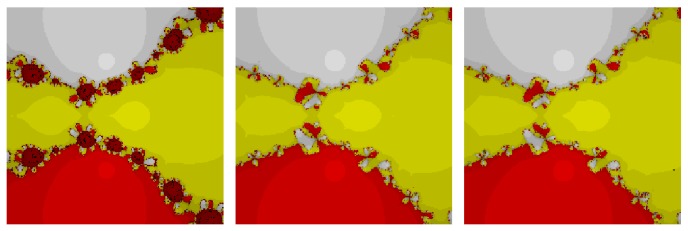
The basins of attraction for SM_8_, OM_8_^1^, and OM_8_^2^, respectively, in problem 3.

*Test Problem 3.* Let *p*_3_(*z*) = (*z* ^3^ + 2*z* − 1), having simple zeros {−0.226699 − 1.46771*i*, −0.226699 + 1.46771*i*, 0.453398}. It is straight forward to see from Figures 5 and 6 that our method, namely, OM_8_^1^ and OM_8_^2^, performed better and larger basins of attraction as compared to the other methods, namely, SSM_8_, LW_8_, TM_8_, and SM_8_. Further, our methods have less number of divergent points as compared to method TM_8_. Note that our methods have also less chaotic behavior as compared to method SM_8_.

## 7. Conclusions

In this paper, we have obtained a simple and elegant families of Ostrowski's method with optimal order of convergence eight by using an additional evaluation of function at the point iterated by Ostrowski's method. Its geometric construction consists in approximating *f*_*n*_′ at *z*_*n*_ in such a way that its average with the known tangent slopes *f*_*n*_′ at *x*_*n*_ and *y*_*n*_ is the same as the known weighted average of secant slopes and then we apply weight function approach. Further, we can also obtain many new optimal families of eighth-order Ostrowski's method by considering different kinds of weight functions which satisfy the conditions mentioned in Theorem 1. Each member of the proposed family requires three evaluations of the function  *f*  and one of its first-order derivative *f*′ per full step and therefore has efficiency index better than fourth-order convergent Ostrowski's method. The superiority of present methods is also corroborated by numerical results displayed in Table 2. Our proposed iterative methods are compared in their efficiency and performance to various other multipoint methods, and it is observed that our proposed methods are efficient and perform better than existing methods available in the literature. Based on Figures 1–6, we conclude that larger basins of attraction belong to our methods, namely, OM_8_^1^ and OM_8_^2^, although the other methods are slow and have darker basins while some of the methods are too sensitive upon the choice of the initial value. Further, this idea can also be extended for the case of King's family.

## Figures and Tables

**Table 1:** Test problems.

*f*(*x*)	*r*	[*a*, *b*]
*f*_1_(*x*) = *x* ^5^ + *x* ^4^ + 4*x* ^2^ − 15	1.3474280989683049815067153807148212	[1.1, 1.6]
$f_{2}\left(x\right)=\sin x-\frac{x}{3}$	2.2788626600758283126999511045618886	[2.0, 3.0]
*f*_3_(*x*) = *e* ^*x*^2^+7*x*−30^ − 1	3.0000000000000000000000000000000000	[2.90, 3.30]
*f*_4_(*x*) = *xe* ^−*x*^	0.0000000000000000000000000000000000	[−1.0, 0.5]
*f*_5_(*x*) = *e* ^−*x*^ + cos⁡*x*	1.7461395304080124176507030889537802	[1.2, 2.5]
*f*_6_(*x*) = 10*xe* ^−*x*^2^^ − 1	1.6796306104284499406749203388379703	[1.5, 1.8]

**Table 2:** Comparison of different eighth-order iterative methods with the same total number of functional evaluations (TNFE = 12).

*f*(*x*)	Initial guess	SSM_8_	LM_8_	TM_8_	SM_8_	MOM1_8_	MOM2_8_
						$b=-\frac{1}{2}$	$b=-\frac{1}{4}$
1	1.1	3.0*e* − 308	4.94*e* − 242	1.1*e* − 156	1.5*e* − 223	7.6*e* − 319	2.3*e* − 314
	1.6	1.9*e* − 340	2.2*e* − 297	1.9*e* − 233	6.7*e* − 297	1.2*e* − 417	4.1*e* − 429

2	2.0	9.83*e* − 386	3.8*e* − 331	3.2*e* − 288	1.7*e* − 339	1.1*e* − 437	1.2*e* − 431
	2.5	9.8*e* − 506	3.6*e* − 462	5.5*e* − 436	1.3*e* − 468	7.7*e* − 556	7.8*e* − 557

3	2.92	1.4*e* − 173	2.8*e* − 92	*D*	1.9*e* − 42	3.0*e* − 177	1.2*e* − 163
	3.20	1.1*e* − 68	2.3*e* − 52	5.6*e* − 27	2.1*e* − 51	8.0*e* − 77	3.0*e* − 77
	3.30	1.8*e* − 28	3.8*e* − 20	1.1*e* − 8	8.3*e* − 20	1.5*e* − 34	3.3*e* − 35

4	−0.5	3.0*e* − 222	1.5*e* − 188	1.5*e* − 139	1.4*e* − 193	1.1*e* − 249	7.8*e* − 252
	0.5	3.5*e* − 123	3.5*e* − 38	*D*	1.1*e* − 3	1.5*e* − 165	3.7*e* − 112

5	1.2	2.2*e* − 516	6.30*e* − 444	1.3*e* − 441	1.3*e* − 456	3.0*e* − 527	1.7*e* − 527
	2.5	6.0*e* − 222	4.5*e* − 157	1.6*e* − 156	7.3*e* − 165	4.5*e* − 266	2.1*e* − 232

6	1.5	4.9*e* − 399	1.5*e* − 351	9.4*e* − 316	1.2*e* − 369	1.4*e* − 477	9.2*e* − 491
	1.8	4.6*e* − 445	3.8*e* − 385	1.0*e* − 325	2.1*e* − 394	5.5*e* − 463	2.2*e* − 461

**Table 3:** Comparison of different eighth-order iterative methods with respect to number of iterations.

*f*(*x*)	Initial guess	SSM_8_	LM_8_	TM_8_	SM_8_	MOM1_8_	MOM2_8_
						$b=-\frac{1}{2}$	$b=-\frac{1}{4}$
1	1.1	3	4	4	4	3	3
	1.6	3	3	4	3	3	3

2	2.0	3	3	3	3	3	3
	2.5	3	3	3	3	3	3

3	2.92	4	4	*D*	4	4	4
	3.30	5	5	5	5	4	4

4	−0.5	4	4	4	4	4	4
	0.5	4	4	*D*	6	4	4

5	1.2	3	3	3	3	3	3
	2.5	3	4	4	4	4	4

6	1.5	3	3	3	3	3	3
	1.8	3	3	3	3	3	3
